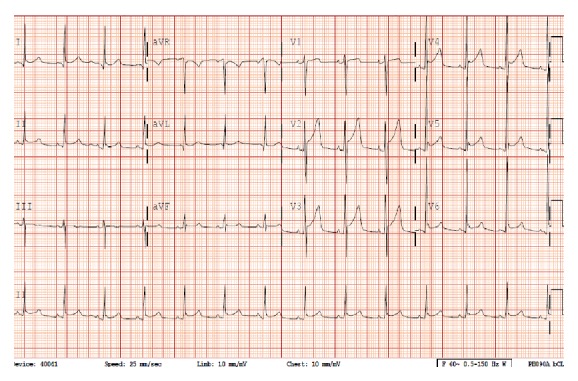# Stabbing Yourself in the Heart: A Case of Autoimmunity Gone Awry

**DOI:** 10.1155/2015/757410

**Published:** 2015-11-19

**Authors:** Hari Vigneswaran, Leslie Parikh, Athena Poppas

**Affiliations:** Warren Alpert Medical School of Brown University, 222 Richmond Street, Providence, RI 02903, USA

## Abstract

Within internal medicine, cardiac and neurologic pathology comprises a vast majority of patient complaints. Physicians and advanced-care practitioners must be highly educated and comfortable in the evaluation, diagnosis, and management of these entities. Chest pain accounts for millions of annual visits to the emergency room with pericarditis diagnosed in approximately four percent of patients with nonischemic chest pain. Guillain-Barre Syndrome is autoimmune polyneuropathy that often results in transient paralysis. Simultaneous diagnosis of both entities is a rare but described phenomenon. Here, we present a clinical case of GBS associated pericarditis. A fifty-five-year-old man with history of renal transplant presented with lower extremity weakness and urinary incontinence. Physical exam and diagnostic studies confirmed Guillain-Barre Syndrome. Patient subsequently developed stabbing chest pain with clinical presentation and electrocardiogram consistent with pericarditis. The patient was successfully treated for both diseases. This case highlights that although infrequent, internal medicine care providers must be cognizant of this correlation to ensure timely diagnosis and treatment.

## 1. Introduction

Guillain-Barre Syndrome is an autoimmune-mediated polyneuropathy. Acute inflammatory demyelinating polyradiculoneuropathy (AIDP) is the most common form of GBS in Western civilization. Clinically, it often presents with areflexic weakness. GBS frequently develops as a postinfectious paralytic process from autoimmune dysregulation. Pericarditis is a disease of inflammation of the fibroelastic sac covering the heart also known as the pericardium. The clinical syndrome results in characteristic positional chest pain with ECG changes. There are many causes of acute pericarditis, including autoimmune dysregulation. Despite this mechanistic similarity, GBS associated pericarditis is a rare phenomenon not commonly described in literature. Here, we present a case report of a patient with Guillan-Barre Syndrome and associated pericarditis.

## 2. Patient Case

A 55-year-old male patient with a history of deceased donor kidney transplant presented to our hospital with lower extremity weakness, gait instability, and urinary incontinence. He reported an antecedent diarrheal illness 2 weeks prior to admission. He was 10 years posttransplant on immunosuppressive agents including tacrolimus, mycophenolate, and low dose prednisone. On physical exam, sensation was decreased below the T11 dermatome. There was decreased vibratory sensation in the distal lower extremities with associated pain in the proximal muscles of the lower extremity. Upper extremity motor strength and reflexes were within normal limits. Lower extremity motor strength however was decreased, with absent patella and ankle jerk tendon reflexes. An infectious lab workup was performed and was negative for adenovirus, parainfluenza, rhinovirus, human-immunodeficiency virus (HIV), and rapid plasma reagin (RPR). Epstein-Barr virus, mycoplasma, and Lyme titers were also within normal range. As the diarrheal episode had occurred two weeks earlier, stool studies were not sent.

Lumbar puncture demonstrated CSF protein seven times the upper limit of normal with few white blood cells. EMG was consistent with axonal demyelination. Guillain-Barre Syndrome was diagnosed based upon these findings as well as the patient's clinical presentation. Treatment was five days of intravenous immunoglobulin. On day five of treatment, the patient developed acute onset chest pain. Pain was stabbing, retrosternal, and positional in nature. ECG was characteristic of pericarditis (Figure 1). The patient began treatment for pericarditis with high dose aspirin and colchicine resulting in symptom improvement. He was subsequently discharged home from the hospital pain-free.

## 3. Discussion

Acute pericarditis is the most common disorder of the pericardium. It is diagnosed in over four percent of patients who present to the emergency room with nonischemic chest pain [1, 2]. The in-hospital mortality rate for pericarditis is 1.1% [3]. The etiology for acute pericarditis is variable including idiopathic, infectious, autoimmune, and malignant origins. Over ninety-five percent of patients present with chest pain [4]. Major clinical criteria include sharp chest pain that is pleuritic in nature and improves upon leaning forward. The presence of a pericardial friction rub is a highly specific but not sensitive finding. Electrocardiogram changes include diffuse widespread ST elevation with PR depression with reciprocal changes in lead aVR. This signifies epicardial inflammation and atrial current of injury [5]. Echocardiogram demonstrates pericardial effusion in approximately sixty percent of patients with acute pericarditis [4].

The classical features of Guillain-Barre Syndrome include ascending symmetric muscle weakness and absent or depressed deep tendon reflexes. Penetrance is variable and can include weakness with mild difficulty ambulating to complete paralysis and respiratory collapse. Less common findings include facial, oculomotor, or upper extremity weakness as well as dysautonomias. GBS usually progresses over two weeks with symptoms starting to abate after one month. Laboratory features include an elevated cerebrospinal fluid protein with a normal white blood cell count. Nerve conduction studies and needle electromyography (EMG) are useful in diagnosis and prognosis.

GBS and pericarditis are two rarely associated diseases. When a patient develops both entities simultaneously, the significance must be determined. In our patient, it was unclear whether the patient's GBS led to pericardial inflammation or if both resulted incidentally from the antecedent virus.

It is known that GBS is usually a postinfectious disorder that involves the formation of autoantibodies that cross-react with gangliosides in the peripheral nerve [6]. This molecular mimicry results in the acute polyneuropathy. We propose that these GBS associated autoantibodies may additionally react with antigens in the pericardium resulting in an autoimmune pericarditis. This mechanism would be similar to rheumatologic autoimmune pericarditis such as lupus induced pericarditis. It is also similar to one proposed etiology of recurrent pericarditis after a primary infectious pericarditis. The recurrent disease is thought to occur from a combination of autoimmune cross-reactivity of the innate immune system targeted at pathogen associated molecular patterns [PAMPs] to toll-like receptors (TLRs) and a failure of dendritic cells to discriminate “self” from microbial “nonself” [4, 7].

In conclusion, this case highlights the importance of recognizing the development of pericarditis when a patient presents with Guillain-Barre Syndrome. Though more research is needed, clinicians should be aware of this association when treating a patient with GBS. This vigilant approach will ensure timely treatment, eradication of pericarditis, and therefore prevention of further complication.

## Figures and Tables

**Figure 1:**